# Nonexercise Equations for Cardiorespiratory Fitness in Older Adults using Body Roundness Index and Waist Circumference

**DOI:** 10.1249/ESM.0000000000000060

**Published:** 2025-12-22

**Authors:** Hayley Chappell, Zachary Gilliam, Bryan Madero, Jenna Springer, Marco Pipoly, Kelsey L. Baller, Chris Oehler, Jeffrey D. Long, Gary L. Pierce, Michelle W. Voss

**Affiliations:** 1 Department of Psychological and Brain Sciences, University of Iowa, Iowa City, IA, USA; 2 Department of Health and Human Physiology, University of Iowa, Iowa City, IA, USA; 3 Interdisciplinary Graduate Program in Neuroscience, University of Iowa, Iowa City, IA, USA; 4 Center for Vital Longevity, School of Behavioral and Brain Sciences, University of Texas at Dallas, Dallas, TX, USA; 5 Department of Psychiatry, University of Iowa, Iowa City, IA, USA; 6 Department of Biostatistics, University of Iowa, Iowa City, IA, USA; 7 Department of Internal Medicine, University of Iowa, Iowa City, IA, USA; 8 Iowa Neuroscience Institute, University of Iowa, Iowa City, IA, USA

**Keywords:** body composition, physical activity, prediction model, resting heart rate

## Abstract

**Introduction:**

Cardiorespiratory fitness (CRF), quantified by oxygen consumption at maximal exercise (VO_2max_), is an important indicator of general health status with aging and related chronic diseases. CRF prediction equations may be an acceptable form of estimating associated health risks when VO_2max_ cannot be measured. The purpose of this study was to describe new CRF prediction equations that utilize waist circumference (WC) and body roundness index (BRI) to improve CRF estimation in inactive older adults.

**Methods:**

Secondary cross-sectional analyses of baseline data from two randomized controlled trials (Exercise Effects on Brain Health and Learning from Minutes to Months [EXTEND], *n* = 113; Effects of a Bicycling Intervention on Cognitive Skills and Cardiovascular Health [BIKE], *n* = 32) included inactive older adults without dementia aged 55–80 yr without psychiatric or cardiovascular diseases. Height, weight, WC, BRI, and body mass index were measured for each participant. Participants also completed the short-form International Physical Activity Questionnaire (IPAQ), the Self-Report Physical Activity Survey (SRPAS), and a maximal exercise test to determine VO_2max_.

**Results:**

Four nonexercise equations estimating CRF were created using linear regression with the variables sex, age, and a combination of IPAQ and WC (eCRF1: *R*^2^ = 0.67, standard error [SE] = 4.18), SRPAS and WC (eCRF2: *R*^2^ = 0.63, SE = 6.49), SRPAS and BRI (eCRF3: *R*^2^ = 0.63, SE = 5.62), or IPAQ and BRI (eCRF4: *R*^2^ = 0.68, SE = 3.46) from EXTEND participants. When validated on out-of-sample BIKE participants, the four new equations were more highly correlated to measured VO_2max_ than previously established models (eCRF1: *R*^2^ = 0.45, constant error [CE] = 0.59; eCRF2: *R*^2^ = 0.42, CE = 1.30; eCRF3: *R*^2^ = 0.47, CE = 1.78; eCRF4: *R*^2^ = 0.52, CE = 0.84).

**Conclusions:**

Equations using WC and BRI perform better for estimating CRF than equations using body mass index and resting heart rate in an inactive older adult population. These equations could be used to screen participants during study enrollment when specific estimates of CRF are desired for the study sample. However, these equations should be validated for use in clinical populations to stratify disease risk.

## INTRODUCTION

As the number of older adults increases, so does the burden of age-related chronic diseases such as cardiovascular disease (CVD), diabetes, Alzheimer's, and related dementias (1). Oxygen consumption at maximal exercise (VO_2max_) is a measure of cardiorespiratory fitness (CRF) and is inversely associated with age-related chronic conditions and all-cause mortality (2,3). For example, relevant to quality of life and financial burden, higher CRF has been associated with reduced dementia risk, lower morbidity following surgery, and reduced length of hospital stays (4,5). An estimation or measurement of CRF can provide both additive and independent information to clinical markers of risk stratification and is a powerful predictor of mortality risk (4,6). This relationship to health outcomes makes CRF measurement clinically meaningful and relevant to aging adults. The gold standard measurement of CRF is the direct measurement of VO_2max_ during a graded maximal exercise test on a treadmill or cycle ergometer. However, because of the physical demands, cost, and lack of accessibility of the equipment required for this test, many clinics cannot offer it. Thus, there is a need to expand and develop nonexercise equations to make measurements of CRF more accessible to wider populations, such as inactive older adults.

In the absence of objectively measured CRF, common variables involved in the estimation of CRF include body mass index (BMI), resting heart rate (RHR), physical activity (PA) status, sex, and age. Previous research suggests that including anthropometric measures such as BMI and waist circumference (WC) as independent variables in equations to predict CRF results in estimates that are comparable to objectively measured CRF (7). Another emerging measure that is more representative of body composition than BMI is the body roundness index (BRI) (8). BRI captures WC in relation to height, modeling the human body shape as an ellipse. The reason BRI and WC may be more predictive than BMI is that CRF is inversely associated with visceral adiposity independent of BMI, reflecting the metabolic advantage of lower visceral adiposity (9). Both WC and BRI are more predictive of visceral adiposity and fat distribution than BMI (8). WC and BRI are also strongly correlated with adverse health risks: individuals with high BRI are at greater risk of CVD incidence, stroke, and cardiac events than individuals with low BRI (10,11). Other existing measures of body composition, such as waist-to-hip ratio and visceral adiposity index, are useful in determining the fat distribution of an individual but have their limitations. Visceral adiposity index is a more complex metric requiring blood-derived lipid markers (12), and waist-to-hip ratio is not as accurate as WC in screening for regional fat distribution (13). Similarly, some equations utilize RHR (14), but RHR is not highly correlated with CRF and is often difficult to measure reliably in clinical and home settings.

Estimating CRF using nonexercise variables is a viable alternative to the direct measurement of CRF. Many estimation equations currently exist in the literature (7,14–18), including popular equations from Jurca et al (14) and Wier et al (7). The equation developed by Jurca et al (14) uses age, sex, BMI, RHR, and self-reported PA to estimate CRF and is commonly used when investigating associations between cognition and brain health in aging (19–22). The equation developed by Wier et al (7) is another accessible equation, which uses age, sex, WC, and self-reported PA (23). Both equations have also been validated in older adult populations (18,24).

PA—any bodily movement produced by skeletal muscle requiring energy expenditure—is a strong behavioral predictor of CRF. Despite the limitations of self-report data, measuring PA levels using questionnaires increases the accessibility of CRF estimates to a wider population. The Self-Report Physical Activity Survey (SRPAS) is one such questionnaire that combines three separate PA questionnaires, resulting in five levels of PA, and is commonly used due to its link with cognition and brain health (14,21). The International Physical Activity Questionnaire (IPAQ) is another common self-report PA questionnaire (18). Compared with SRPAS, IPAQ has been evaluated more often for reliability and validity and is more widely used, increasing its accessibility for use in nonexercise equations. However, IPAQ’s current categorization of PA is limited because it calculates metabolic equivalents of task minutes (MET-min) from self-reported PA and categorizes activity into three levels. As such, categorization cutoffs are arbitrary and not based on health benefits or PA guidelines (25), and the three PA levels do not provide enough granularity to characterize individual differences or motivate incremental behavioral changes between categories.

Although PA is an essential part of a healthy lifestyle (25), CRF cannot be estimated from subjectively measured PA alone. This is especially important for inactive older adult populations, in which CRF estimations may help assess adverse health risks if self-reported PA is otherwise low and access to physician-supervised maximal exercise tests is a barrier to CRF assessment. Other factors, such as sex, age, anthropometric measures, and genetics, play a large role in any person’s baseline CRF, with or without participation in PA. We aimed to determine whether WC and BRI are equally or more effective and reliable at predicting CRF than BMI and RHR. Additionally, we sought to establish an enhanced categorization of PA for IPAQ application in nonexercise CRF equations specifically designed for an older adult population.

## METHODS

### Participants

Participants were drawn from two randomized controlled trials, Exercise Effects on Brain Health and Learning From Minutes to Months (EXTEND; NCT03114150) and Effects of a Bicycling Intervention on Cognitive Skills and Cardiovascular Health (BIKE; NCT02453178). Inclusion criteria were age ranging from 55 to 80 yr (EXTEND) or 60 to 80 yr (BIKE) and being from Iowa City, IA, USA, and surrounding areas. Inclusion criteria specific to the trials were not participating in moderate-intensity PA greater than 60 min/wk for the past 6 months (EXTEND) and not participating in moderate-intensity PA for more than 30 min in 1 d on any more than 3 d·wk^−1^ (BIKE). Exclusion criteria common across the two studies included the following: 1) inability to comply with experimental instructions; 2) qualifying as “high risk” for acute cardiovascular events based on published standards from the American College of Sports Medicine; 3) diagnosis of depression, attention-deficit disorder or attention-deficit/hyperactivity disorder, epilepsy, meningitis, Parkinson disease, a heart condition or other cardiovascular event, chronic obstructive pulmonary disease, uncontrolled asthma (not on medication or inhaler for the past 3 months or more), cystic fibrosis, unregulated thyroid disorder (not on medication for the past 3 months or more), renal or liver disease, or heart murmur; having brain surgery; and smoking or living with someone who smokes in the past 3 months. Exclusion criteria specific to EXTEND were as follows: 1) score <20 on the Montreal Cognitive Assessment; 2) taking antipsychotic medications; 3) inability to complete magnetic resonance imaging (e.g., due to magnetic implants that would preclude magnetic resonance imaging, such as an aneurysm clip or metallic stent, metallic fragments in the body, or cardiac pacemaker or other electronic implant or device); 4) diagnosis of Alzheimer's disease, bipolar disorder, dissociative disorder, claustrophobia, stroke, human immunodeficiency virus/acquired immunodeficiency syndrome, or colorblindness. Exclusion criteria specific to BIKE were as follows: 1) score ≤24 on the Mini-Mental State Exam, 2) diagnosis of anxiety disorder, stroke, or head injury.

Only baseline data from EXTEND and BIKE were used for the current analysis, and all data were collected at the time of study visits. Of the 122 total participants in EXTEND, 113 participants with all required baseline data were included in the analysis. Required baseline data included the completion of a preintervention maximal exercise test, WC measurement from the first week of the intervention, and the completion of either the IPAQ or SRPAS. Of the 113 EXTEND participants included in the analysis, 58 completed the SRPAS. The BIKE study had a smaller sample size and 32 participants had all the required baseline data. Written informed consent was obtained before participation, and ethical approval was granted by the University of Iowa Institutional Review Board (EXTEND: IRB ID# 201705800; BIKE: IRB ID# 201405837).

### Exercise Testing

Participants in both study samples completed a symptom-limited maximal exercise test to exhaustion on an upright cycle ergometer (Lode BV, Groningen, the Netherlands) with 12-lead electrocardiography and breath-by-breath cardiopulmonary gas analysis (True One; Parvomedics, Inc., Salt Lake City, UT, USA) for determining peak oxygen consumption (VO_2peak_) supervised by a trained exercise specialist and licensed cardiologist. RHR in an upright position was collected during the visit using a Polar chest-worn heart rate sensor (Polar Electro Oy, Kempele, Finland). Both studies followed a standardized ramp protocol that increased resistance every 2 min. The purpose of this test was to identify each participant’s CRF operationalized as their VO_2peak_ in mL·kg^−1^·min^−1^, determined by whether two of the following three criteria were met: respiratory exchange ratio value of ≥1.10, rating of perceived exertion of ≥17 (on 6–20 Borg scale) (26), or maximal heart rate within 10 bpm of age-predicted (based on the following formula: 220 − age) maximal heart rate (or attainment of 80% of predicted heart rate maximum or greater).

### Self-Reported Physical Activity

**Figure 1.**
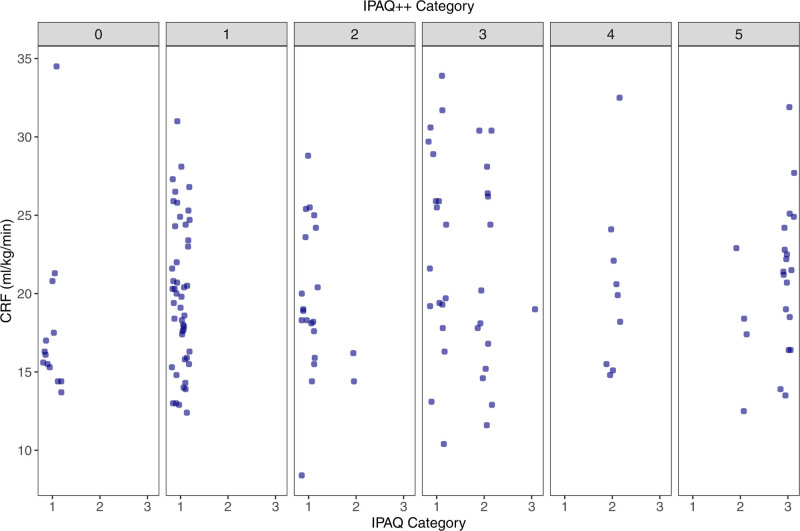
Distribution of participants categorized by the International Physical Activity Questionnaire (IPAQ) versus our adjusted IPAQ (IPAQ^++^) categories and measured cardiorespiratory fitness (CRF). Scatterplots within each column show the relationship between the three IPAQ categories (bottom) and IPAQ^++^ categories (top) and CRF (*y*-axis) in all participants (*n* = 145).

Both study samples completed an initial visit that included informed consent and a health history questionnaire that collected information on demographics, personal and family medical history, and health behaviors. Of this information, the responses to the IPAQ and SRPAS questions used by Jurca et al (14) were used for the current analyses (27). SRPAS was added to the battery of surveys halfway through the completion of EXTEND as a method to estimate CRF during coronavirus disease 2019 (COVID-19) restrictions when in-person testing was not possible, resulting in roughly half of EXTEND participants completing the survey. Answers from the IPAQ were categorized in two ways: 1) following standard recommendations for the IPAQ short form used by Sewell et al (18,27), yielding three categories, and 2) using moderate–to-vigorous PA (MVPA) thresholds based on accelerometer-derived activity minutes, yielding six categories, henceforth named IPAQ^++^ (28). Figure 1 plots the distribution of participants categorized by IPAQ versus IPAQ^++^ and their respective measured CRF. The benefits of categorizing IPAQ into six levels based on PA-minute thresholds include increased granularity, use of minutes of MVPA instead of MET-min, inclusion of walking-related activity via self-reported MVPA, and greater association with health outcomes (28). Table 1 outlines the thresholds and scoring criteria for each of the self-report measures for PA assessment.

**Table 1:** Scoring of Self-Reported Measures of Physical Activity.

Questionnaire	Reported Answer	Score
IPAQ	<600 total MET-min per week	1
	480–1500 vigorous or 600–1500 total MET-min per week	2
	>1500 vigorous or >3000 total MET-min per week	3
IPAQ^++^	0 min of moderate-to-vigorous activity per week	0
	1–75 min of moderate-to-vigorous activity per week	1
	76–150 min of moderate-to-vigorous activity per week	2
	151–300 min of moderate-to-vigorous activity per week	3
	301–500 min of moderate-to-vigorous activity per week	4
	>500 min of moderate-to-vigorous activity per week	5
SRPAS	1: inactive or little activity other than usual daily activities	0
	2: Regularly (≥5 d·wk^−1^) participate in physical activities requiring low levels of exertion that result in slight increases in breathing and heart rate for at least 10 min at a time	0.32
	3: Participate in aerobic exercises, such as brisk walking, jogging or running, cycling, swimming, or vigorous sports at a comfortable pace or other activities requiring similar levels of exertion for 20–60 min·wk^−1^	1.06
	4: Participate in aerobic exercises, such as brisk walking, jogging, or running at a comfortable pace or other activities requiring similar levels of exertion for 1–3 h·wk^−1^	1.76
	5: Participate in aerobic exercises, such as brisk walking, jogging, or running at a comfortable pace or other activities requiring similar levels of exertion for over 3 h·wk^−1^	3.03

IPAQ and IPAQ^++^ group activity into ordinal categories, which were used in the regression equations. SRPAS assigns a physical activity weight created from linear regression to each option 1–5.

IPAQ, International Physical Activity Questionnaire; IPAQ^++^, IPAQ with adjusted categories; MET-min, metabolic equivalent minutes; SRPAS, Self-Report Physical Activity Survey.

### Anthropometric Measures

Height and weight were collected using a calibrated physician’s scale (Detecto, Las Vegas, NV, USA) by a trained exercise specialist during the health measures visit in both studies. WC was measured in inches at the level of the umbilicus but converted into centimeters for analyses. This measurement was collected at multiple points during each study; however, self-reported WC measurements from week 1 of each study intervention were used for analyses due to missing data at baseline. High correlation was seen between the existing baseline and week 1 measurements (EXTEND full *n* = 78, *r* = 0.93, *P* < 0.001; EXTEND subset *n* = 23, *r* = 0.96, *P* < 0.001; and BIKE *n* = 30, *r* = 0.93, *P* < 0.001). Height and weight were used to calculate BMI, and height and WC were used to calculate BRI using the following formula (8):


$$\text{BRI}=364.2-365.5\times \sqrt{1-\left(\frac{{\left(\frac{\text{WC}}{2\pi }\right)}^{2}}{{\left(\frac{\text{Height}}{2}\right)}^{2}}\right)}$$

### Statistical Analysis

**Figure 2.**
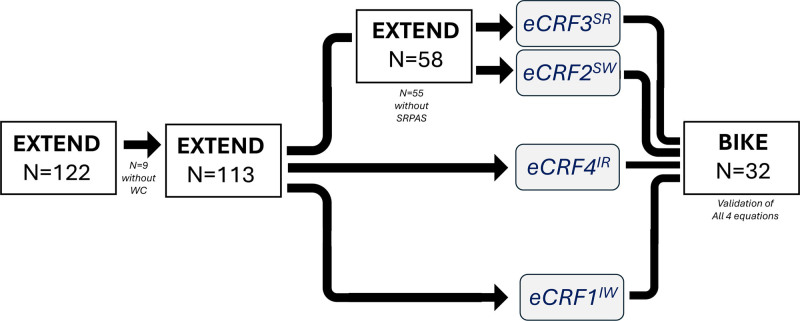
Groupings of participants for the derivation and validation of four cardiorespiratory fitness (CRF) estimation equations (eCRF). The four CRF estimation equations used Exercise Effects on Brain Health and Learning From Minutes to Months (EXTEND) participants as the derivation sample and Effects of a Bicycling Intervention on Cognitive Skills and Cardiovascular Health (BIKE) participants as the validation sample. All equations had the common variables of age and sex. Superscript letters are used to indicate which anthropometric measure and physical activity survey were used in each respective equation. I, International Physical Activity Questionnaire with adjusted categories; R, body roundness index; S, Self-Report Physical Activity Survey (SRPAS); W, waist circumference (WC).

All analyses were conducted using jamovi version 2.6.2 (jamovi project, Sydney, Australia) — graphical user interface for R — and associated packages (29,30). Figure 2 illustrates the process of deriving and validating the four CRF estimation equations. The first step was to use data from EXTEND to derive the four equations using linear regression. Independent variables were sex, age, and either IPAQ and WC, SRPAS and WC, SRPAS and BRI, or IPAQ and BRI; baseline CRF measured as VO_2max_ in mL·kg^−1^·min^−1^ was the dependent variable. The full EXTEND sample (*n* = 113) was utilized for the two equations using IPAQ, and a subset of EXTEND (*n* = 58) was utilized for equations using SRPAS. For all equations, sex was dummy-coded (0 = female, 1 = male) as is common practice (7,14–18). Each equation was separately tested to estimate the CRF for participants in each sample.

To examine and compare the performance of our four new equations against widely used CRF measures, we also estimated CRF in all EXTEND and BIKE participants using the Jurca et al (14) and Wier et al (7) equations. Of note, the equation used in this analysis from Wier et al (7) was modified by Sewell et al (18), replacing the use of the US National Aeronautics and Space Administration/Johnson Space Center PA scale with the IPAQ while keeping the original equation’s coefficients the same. We used the Sewell et al (18) construction of Wier et al (7) since the former validated the equation on a cognitively unimpaired sample of older adults.

*T* tests, grouped by study (EXTEND vs BIKE), were conducted for all continuous variables to identify any differences across EXTEND and BIKE participants. Skewness was calculated for self-reported PA minutes from IPAQ. Pearson’s *r*, *R*^2^, constant error (CE), and mean absolute percent error (MAPE) for each equation were examined to rank performance. Each equation’s CE was measured as the average difference between the measured and estimated CRF. A positive CE value indicates an underestimation of CRF, whereas a negative CE value indicates an overestimation of CRF. The smallest average difference between measured and estimated CRF, or CE close to 0, supports the accuracy of the equation. The standard deviation (SD) of CE indicates how much CE varies from the average across different data points. MAPE, another measure of prediction accuracy, describes the percent error of an equation, where a lower percentage portrays higher accuracy. Additionally, the Akaike Information Criterion (AIC) was estimated for the estimated CRF equations we derived. AIC is a measure of model quality that balances goodness of fit with model complexity. A lower AIC indicates a better fit, and values help compare multiple regression models to evaluate which model best explains the data without overfitting. Bland–Altman plots were created for each equation to visualize the mean differences between estimated and measured CRF and the 95% confidence intervals. These plots were intended to indicate any patterns of error within the estimation equations. Other descriptive figures visualized the distribution of participants within IPAQ categories compared with IPAQ^++^ categories.

## RESULTS

Descriptive characteristics for EXTEND (*n* = 113) and BIKE (*n* = 32) participants are provided in Table 2. A total of 13 participants out of the total sample did not meet the criteria for a valid assessment of VO_2peak_. Descriptive characteristics for the full EXTEND sample (*n* = 113) include participants used to derive eCRF1 and eCRF4; descriptive characteristics for the EXTEND subset of *n* = 58 were participants with SRPAS who were used to derive eCRF2 and eCRF3 (see Table 2 and Fig. 2). Participant characteristics in each study are similar. After running *t* tests using study as the grouping variable, we observed no significant differences between EXTEND and BIKE participants in BMI, BRI, WC, baseline CRF, or PA level from SRPAS (all *P* > 0.05). There were significant differences in age (*P* < 0.001), RHR (*P* = 0.002), and PA level from IPAQ (*P* < 0.001). The BIKE sample was older, with higher RHR and higher self-reported activity from IPAQ. PA was positively skewed for both MET-min (skewness = 4.79) and PA minutes from IPAQ (skewness = 5.76); this did not impact analyses, given our conversion to ordinal groupings of PA levels as shown in Figure 1. The percentage of males versus females and the distribution of participants across 5-year age increments can be seen in Supplemental Digital Content 1 (figure, https://links.lww.com/EM9/A47).

**Table 2:** Descriptive Characteristics of the Study Participants.

	EXTEND				BIKE	
	Full (*n* = 113)		Subset (*n* = 58)		Full (*n* = 32)	
Variables	Male (*n* = 38)	Female (*n* = 75)	Male (*n* = 14)	Female (*n* = 44)	Male (*n* = 12)	Female (*n* = 20)
Age, yr^*a*^	63.8 ± 6.2	63.1 ± 5.7	62.9 ± 6.0	63.2 ± 5.4	68.6 ± 5.5	66.5 ± 3.4
MoCA	26.6 ± 26.6	27.6 ± 1.7	26.6 ± 1.9	27.5 ± 1.8	27.3 ± 1.5	27.6 ± 1.8
Education, yr	17.1 ± 2.4	16.4 ± 2.3	17.5 ± 2.3	16.7 ± 2.2	17.1 ± 3.7	17.9 ± 1.8
Waist circumference, cm	103.9 ± 12.6	95.6 ± 14.2	101.3 ± 13.0	92.8 ± 13.9	112.0 ± 16.6	96.5 ± 13.6
Body mass index, kg·m^−2^	29.9 ± 4.9	29.5 ± 5.7	28.9 ± 4.0	28.2 ± 5.6	31.4 ± 5.1	28.1 ± 5.6
Body roundness index	5.2 ± 1.7	5.1 ± 1.9	5.0 ± 1.6	4.7 ± 1.8	6.6 ± 2.2	5.4 ± 1.9
Resting heart rate, bpm^*a*^	74.6 ± 12.0	79.9 ± 12.0	75.6 ± 10.7	78.9 ± 10.7	72.9 ± 12.1	69.4 ± 9.3
IPAQ MET-min^*a*^	465.0 ± 941.0	273.0 ± 498.0	558.0 ± 1066.5	225.0 ± 1411.5	1273.0 ± 2894.0	917.0 ± 2708.0
IPAQ PA minutes^*a*^	125.00 ± 200.00	70.00 ± 120.00	150.00 ± 251.25	60.00 ± 105.00	383.00 ± 589.00	273.00 ± 716.00
SRPAS, 1–5	2	2	2	2	1	2
Baseline VO_2peak_, mL·kg^−1^·min^−1^	24.9 ± 5.3	18.2 ± 3.9	24.2 ± 5.2	18.5 ± 4.5	22.1 ± 5.1	18.0 ± 4.1

Data are shown as mean ± standard deviation. IPAQ MET-min and PA minutes (total physical activity minutes) are totals per week, reported as median ± interquartile range. SRPAS data are shown as mode. EXTEND full (*n* = 113) describes the sample used for cardiorespiratory fitness estimation equation 1 (eCRF1) and eCRF4. EXTEND subset (*n* = 58) describes the sample used for eCRF3 and eCRF4. BIKE (*n* = 32) describes the sample used for out-of-sample validation of all equations. Sex-specific differences exist in waist circumference, resting heart rate, and baseline CRF (all *P* < 0.05). The Montreal Cognitive Assessment (MoCA), a tool for the detection of mild cognitive impairment, is scored out of 30 total points. Waist circumference is the value self-reported by participants at week 1 of the intervention. The self-reported values were strongly correlated with the values obtained by a trained exercise specialist at baseline for the EXTEND full, *n* = 78 (*r* = 0.93, *P* < 0.001), EXTEND subset, *n* = 23 (*r* = 0.96, *P* < 0.001), and BIKE, *n* = 30 (*r* = 0.93, *P* < 0.001) groups.

^*a*^Variables showed significant differences between EXTEND and BIKE participants.

BIKE, Effects of a Bicycling Intervention on Cognitive Skills and Cardiovascular Health; EXTEND, Exercise Effects on Brain Health and Learning From Minutes to Months; IPAQ, International Physical Activity Questionnaire; MET-min, metabolic equivalent minutes; SRPAS, Self-Report Physical Activity Survey; VO_2peak_, peak oxygen consumption.

Table 3 lists the four CRF equations resulting from linear regression in addition to the two equations used for comparison. As expected, the estimated CRF derived from all equations was significantly correlated with the measured CRF in the same sample (*P* < 0.001). Supplemental Digital Content 2 (table, https://links.lww.com/EM9/A48) includes the standardized *β* coefficient and the 95% confidence interval for each variable used in the CRF estimation equations from Table 3. Each equation was recreated to include only participants who completed SRPAS (see Supplemental Digital Content 3, table, https://links.lww.com/EM9/A49), and the results from each equation were comparable (see Supplemental Digital Content 4, table, https://links.lww.com/EM9/A50).

**Table 3:** Equations for Estimating Cardiorespiratory Fitness CRF (eCRF) From Current Analyses With EXTEND Sample and Established Authors.

Author	Equation	*r*	*R* ^2^	SEE	*n*
eCRF1^IW^	56.05 − 0.294*Age + 8.712*(Sex) − 0.205*WC + 0.123*IPAQ^+ +^	0.82	0.67	4.18	113
eCRF2^SW^	55.288 − 0.281*Age + 7.270*Sex − 0.216*WC + 0.547*SRPAS	0.79	0.63	6.49	58
eCRF3^SR^	43.576 − 0.285*Age + 5.824*Sex − 1.723*BRI + 0.563*SRPAS	0.79	0.63	5.62	58
eCRF4^IR^	43.95 − 0.284*Age + 7.092*Sex − 1.550*BRI + 0.062*IPAQ^++^	0.83	0.68	3.46	113
Wier et al (7) ^IW^	59.416 − 0.327*Age + 11.488*Sex − 0.266*WC + 1.297*IPAQ	0.81	0.66	4.80	2801
Jurca et al (14) ^SM^	(18.07 − 0.10*Age + 2.77*Sex − 0.17*BMI − 0.03*RHR + SRPAS)*3.5	0.81	0.66	5.08	1863

Reported Pearson's *r*, *R*^2^, and the standard error of the estimate (SEE) in mL·kg^−1^·min^−1^ are as listed by the original author for the two comparison equations and from the original EXTEND derivation group for the four eCRF. Age is in years. Sex is male = 1 or female = 0. Superscript letters are used to indicate which anthropometric measure and physical activity survey were used in each respective equation. M indicates BMI; R, BRI; I, IPAQ^++^; S, SRPAS; W, WC.

BMI, body mass index; BRI, body roundness index; EXTEND, Exercise Effects on Brain Health and Learning From Minutes to Months; IPAQ, International Physical Activity Questionnaire in three categories; IPAQ^++^, IPAQ adjusted to six categories; RHR, resting heart rate (bpm); SRPAS, Self-Report Physical Activity Survey; WC, waist circumference.

Outlined in Table 4 are the results when the four derived CRF equations and two comparison equations were validated on the out-of-sample BIKE participants. Pearson’s *r*, *R*^2^, CE, and MAPE data from Table 4 suggest that eCRF4 outperformed all other equations. AIC values, calculated for all our derived CRF estimation equations, show that eCRF4 achieved the best balance between model fit and complexity without including unnecessary parameters compared with the other three equations (Table 4). Additionally, when comparing all CRF equations together, our equations have a greater Pearson’s *r* and *R*^2^ than the equation from Jurca et al (14). Each of our CRF equations also has a lower absolute CE, SD, and MAPE than the equation from Wier et al (7).

**Table 4:** Comparison of the Cardiorespiratory Fitness Estimation Equations (eCRF) When Validated on BIKE Participants.

	*r* (LL, UL)	*R* ^2^	CE ± SD	MAPE ± SD (%)	AIC
eCRF1^IW^	0.67 (0.43, 0.83)	0.45	0.59 ± 7.05	16.24 ± 0.14	590.2
eCRF2^SW^	0.65 (0.39, 0.81)	0.42	1.30 ± 7.20	17.11 ± 0.14	311.5
eCRF3^SR^	0.69 (0.44, 0.83)	0.47	1.78 ± 6.99	17.57 ± 0.12	587.6
eCRF4^IR^	0.72 (0.49, 0.85)	0.52	0.82 ± 6.58	15.31 ± 0.12	311.1
Wier et al (7) ^IW^	0.67 (0.42, 0.83)	0.45	2.25 ± 8.50	20.48 ± 0.17	-
Jurca et al (14) ^SM^	0.63 (0.36, 0.81)	0.34	−0.78 ± 9.76	21.17 ± 0.25	-

Estimation equations were validated on BIKE participants (*n* = 32). Pearson's *r* (lower limit [LL], upper limit [UL]) and *R*^2^ for all equations are reported after validation on BIKE participants. Constant error (CE) = Σ(measured VO_2max_ − estimated VO_2max_)/*n*. Mean absolute percent error (MAPE) = (1/*n*)*Σ( measured VO_2max_ − estimated VO_2max_ / measured VO_2max_ )*100. AIC is a measure of model quality that balances goodness of fit with model complexity (lower is better). Superscript letters are used to indicate which anthropometric measure and physical activity survey were used in each respective equation.

AIC, Akaike Information Criterion; BIKE, Effects of a Bicycling Intervention on Cognitive Skills and Cardiovascular Health; I, International Physical Activity Questionnaire adjusted to six categories; M, body mass index; R, body roundness index; S, Self-Report Physical Activity Survey; SD, standard deviation; VO_2max_, maximal oxygen consumption; W, waist circumference.

**Figure 3.**
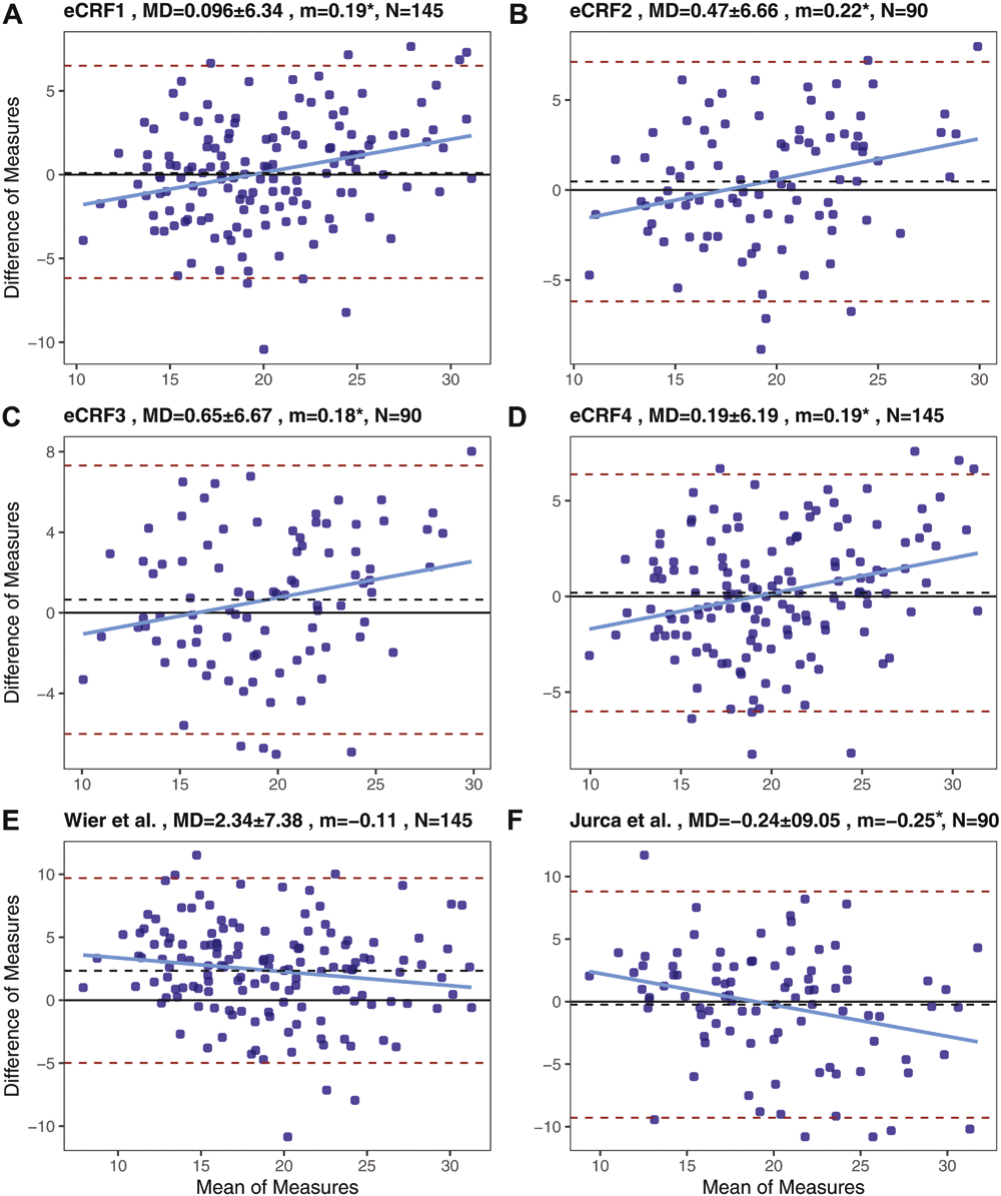
Bland–Altman plots for each equation between estimated and measured cardiorespiratory fitness (CRF). A. Estimated CRF equation 1 (eCRF1). B. eCRF2. C. eCRF3. D. eCRF4. E. Equation from Wier et al (7). F. Equation from Jurca et al (14). m, slope of the regression line; MD, mean difference between estimation equation and measured CRF ± standard deviation. *Slope of the regression line is significantly different than 0.

Additionally, as seen in Figure 3, Bland–Altman plots were created for each CRF equation including data from EXTEND and BIKE participants. These plots assess the agreement between measured CRF and estimated CRF and identify any bias between the two measures. The Bland–Altman plots of the four equations from this analysis show higher agreement compared with the performance of the equations from Wier et al (7) and Jurca et al (14), as evidenced by the low mean differences and small SD. Additionally, plots from Wier et al (7) and Jurca et al (14) estimations showed more data points near or outside the 95% confidence interval, indicating that there was more unexplained error than in our four CRF estimation equations. Bias is noted in our four CRF equations: at lower CRF, the equations tend to overestimate CRF, whereas at higher CRF, the equations tend to underestimate. The opposite is seen for the two equations used for comparison.

## DISCUSSION

Our results demonstrate that estimating CRF with nonexercise equations that use WC or BRI and six categories of PA minutes from IPAQ had out-of-sample predictive validity in an inactive older adult sample better than or very comparable to existing equations (7,14–16,18). Specifically, eCRF4, using BRI and IPAQ^++^, performed better than our other CRF equations as well as Jurca et al (14) and comparably to the equation from Wier et al (7). Our four CRF equations also accurately estimated CRF percentiles, adjusted by demographics, as seen in Supplemental Digital Content 5 (figure, https://links.lww.com/EM9/A51), proving them valuable for potential use in both clinical and research settings.

PA is a strong predictor of CRF, but it is unclear which self-report measure of PA is best for predicting CRF. Besides SRPAS and IPAQ, some groups use the National Aeronautics and Space Administration PA scale, the Ball State PA scale, or the Trøndelag Health Study questionnaire (7,14–18,23). Additionally, the current categorization of PA levels in the IPAQ for use in nonexercise-based CRF equations is limited. After comparing how participants were categorized in our IPAQ^++^ versus IPAQ, we saw there was increased granularity to differentiate PA levels and a stronger correlation with objectively measured CRF when using IPAQ^++^ categories. The increased granularity from IPAQ^++^ categories helped to separate and mitigate effects from skewed distributions and outliers. Participants reporting extreme values are not grouped together with those who report more moderate to high levels of activity, and at the same time, extreme PA will not result in overestimation of CRF. Moreover, IPAQ^++^ categories are based on accelerometer-derived MVPA thresholds that are associated with lower disease risk (28). In an applied setting, participants can relate moving up in an IPAQ^++^ category with predicted increases in CRF and decreased risk of disease, allowing for improved understanding of the positive impact of PA. Our results support the claim that IPAQ^++^ outperforms SRPAS. This is observed in eCRF1 and eCRF4, which have CE closer to 0, smaller SD, and greater *r* and *R*^2^ than the rest of the equations. SRPAS is simple to use in the field with brief statements participants can choose from, yet it does not directly measure PA minutes and has not been validated with health outcomes like IPAQ. On the other hand, although the IPAQ measure of MET-min more directly measures PA minutes, it is not as easy for participants to fill out independently and leaves more room for recall bias toward the most active days of the week, rather than capturing true behavior within a full week. In the future, more descriptive examples of PA intensity should be presented to participants when they complete PA surveys; we suspect outliers in reported activity occur when participants do not fully understand the meaning of MVPA or which activities count as PA.

Relationships between proxies of body composition and CRF are well-investigated (31). Equations utilizing WC as a measure of body fat distribution outperformed the comparison equation using BMI and RHR, and equations utilizing BRI outperformed both, as evidenced by greater *R*^2^ and smaller CE and standard error of the estimate. Our four CRF equations address an issue reported by Jackson et al (15): although including a direct measurement of percent body fat in estimation equations would increase the accuracy of estimated CRF, it is not a measure that would be feasible for mass testing or at-home use. WC and BRI are cost-effective measures for both clinical and research settings that could be easily assessed through self-report. Assessing visceral adiposity increases WC and BRI sensitivity to the distribution of body fat, which is why these measures are more predictive of body composition than BMI (32). BMI is also not as strongly correlated with cardiometabolic disease or risk factors as BRI and WC. Previous studies show that BRI can significantly indicate the presence of metabolic dysfunction or insulin resistance and predict risk for CVD and all-cause mortality (10,11). WC is already established as a criterion for metabolic syndrome and is associated with higher mortality risk, independent of BMI (32,33). The equations from this analysis would allow researchers and clinicians to apply more accurate measures of body composition to estimate CRF. Additionally, our equations are applicable to community use because they are based on self-reported WC from participants and do not require a trained professional for measurement. Participants and patients alike could accurately estimate CRF from outside of a controlled research setting. Unfortunately, the measurement of WC is not as widespread as BMI, which may be a limitation.

The results of this analysis should be interpreted in the context of the following limitations. Both EXTEND and BIKE exhibited homogeneity in ethnicity and baseline CRF within an older adult sample. This makes our equations specific to inactive older adults who identify as White and non-Hispanic. Although ethnicity is not a significant contributing variable of CRF, data from Wier et al (7) underscore the issue that equations developed from participants who identify as White and non-Hispanic are less accurate when used to predict CRF for non-White participants. The effect of demographic differences between ethnic groups on body composition may play a role in this discrepancy; data from the National Health and Nutrition Examination Survey 2015–2016 show WC was highest in men identifying as non-Hispanic White and women identifying as non-Hispanic Black, whereas WC was lowest in both men and women identifying as Asian (34). However, the mechanism behind these differences is still unclear (35). Another potential limitation is that our study did not intentionally sample across a full spectrum of body composition variables within our age range. However, as shown in Supplemental Digital Content 6 (table, https://links.lww.com/EM9/A52), the distribution of age across BMI and BRI categories is relatively stable. Our sample also exhibited outliers in self-reported PA from IPAQ, causing the distribution to be skewed. Although self-report measures have more outliers and errors from participants, the accessibility they offer outweighs these risks. Finally, only *n* = 58 of the participants in EXTEND completed the SRPAS due to a change in protocol during COVID-19. This resulted in our four equations having varying sample sizes used for linear regression, possibly contributing to differences in effect sizes. Supplemental Digital Content 3 (table, https://links.lww.com/EM9/A49) and Supplemental Digital Content 4 (table, https://links.lww.com/EM9/A50) contain data from the linear regression of all four equations with the sample size consistent (*n* = 58), and the results did not meaningfully change conclusions.

Future work should examine the association between our equations and health biomarkers and outcomes associated with CRF among older adults, such as cognitive performance and brain structure and function. Both empirical and meta-analytic review studies support that increasing CRF among older adults can enhance cognitive function and slow brain aging (21,36). This is particularly relevant to this analysis because the four new CRF equations were derived and validated on an older adult sample. Further considerations include testing these equations in exercise intervention studies to empirically validate the extent to which changing PA levels result in changes in CRF to the levels predicted by the equations. Results from this analysis show that the new equations are accurate and valid in estimating baseline CRF; however, it would be important to know if these equations remain accurate when predicting changes in CRF after an exercise intervention. Additional research should investigate using other measures of body composition to estimate CRF, such as A Body Shape Index, which is predictive of visceral adiposity and body proportions and has not been used as a variable in equations to estimate CRF to our knowledge (37,38).

The proposed equations from our study extend the existing nonexercise equations for predicting CRF by replacing BMI and RHR with WC or BRI, as well as implementing an improved way to categorize PA minutes from IPAQ. According to our results, estimated CRF from our four nonexercise equations would be useful in either clinical or research settings to investigate the effects of CRF on health outcomes in older adult populations when maximal exercise testing and direct measurement of VO_2max_ are not feasible.

## ACKNOWLEDGMENTS

We thank Rachel Cole, Timothy Weng, Ellie Henry, Jillian Kousins, Conner Wharff, Chase Hamilton, Abby O’Deen, Will Daniels, Lyndsey DuBose, Kristen Davis, Abbi Lane, Matthew Armstrong, Colin Gimblet, Amy Stroud, Michael Muellerliele, Nagalakshmi Nagarajan, Grace Foster, Thorarinn Bjarnason, Nidal Harb, and Lauren Reist on the Bike EXTEND study team for their help with data collection.

The results of the present study do not constitute endorsement by the American College of Sports Medicine. The results of the study are presented clearly, honestly, and without fabrication, falsification, or inappropriate data manipulation.

## CONFLICTS OF INTEREST AND SOURCE OF FUNDING

None of the authors have any conflicts of interest to report. This work was supported by the National Institute of General Medical Sciences (T32GM108540) and the National Institute on Aging (R21AG048170 and R01AG055500). G.L.P. is supported by the Russell B. Day and Florence D. Day Chair in Liberal Arts and Sciences at the University of Iowa.

## DATA AVAILABILITY

The datasets generated and/or analyzed during the current study are available here: https://doi.org/10.17605/OSF.IO/CX6P3.

## Supplementary Material


