# Supplementary material for: Nonexercise Equations for Cardiorespiratory Fitness in Older Adults using Body Roundness Index and Waist Circumference
Source: Exerc Sport Mov. 2025 Dec 22;4(1):e00060. doi: 10.1249/ESM.0000000000000060 (PMC12721680; doi:10.1249/ESM.0000000000000060)
Supplement: Supplementary file 1 [file esam-4-e00060-s001.docx]

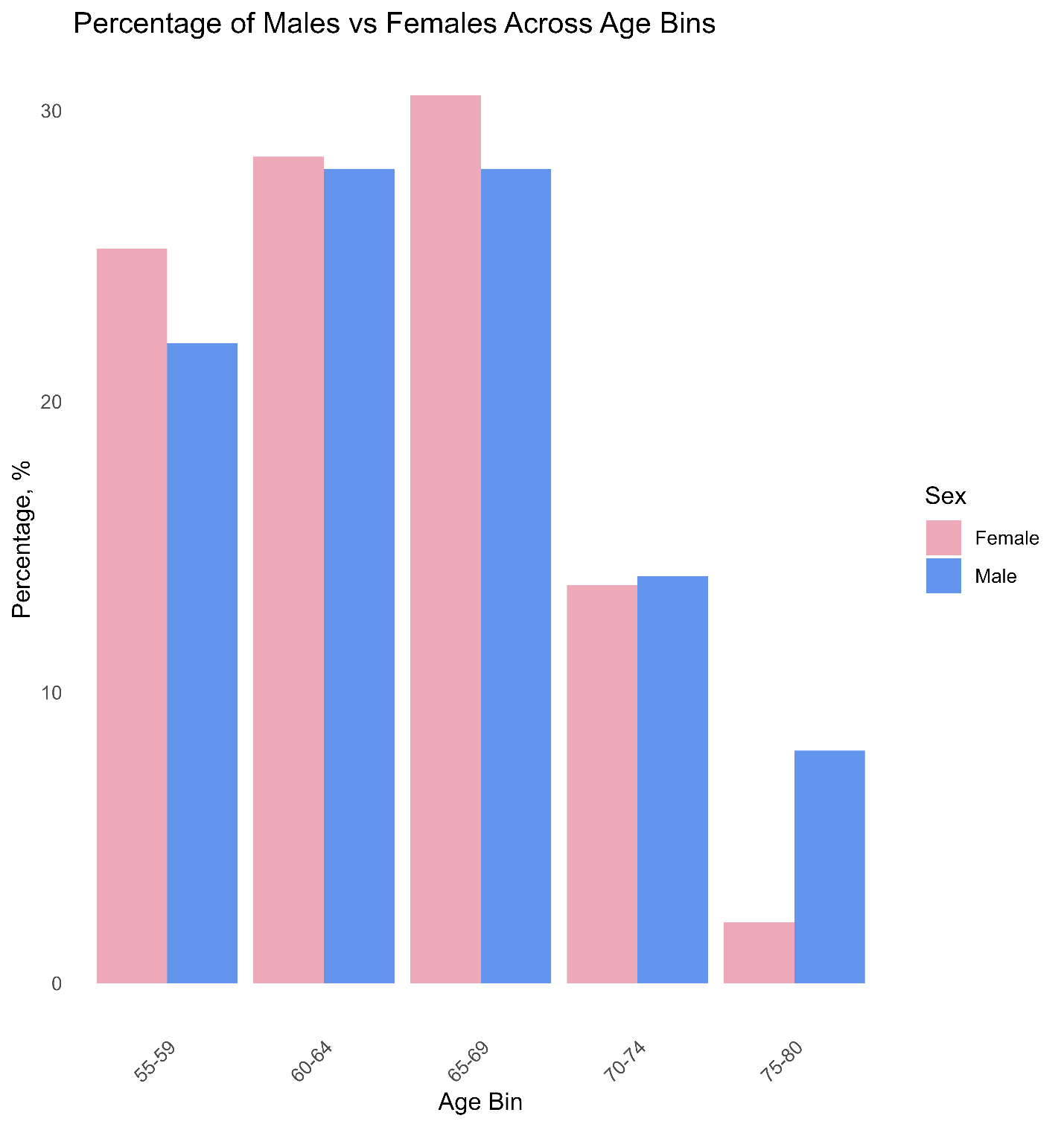


**Supplemental Content 1.** The percentage of males vs females per age bin is calculated as the number of males in a certain age bin out of the total number of males in the sample (n=50) and the number of females in a certain age bin out of the total number of females in the sample (n=95).
