# Supplementary material for: Nonexercise Equations for Cardiorespiratory Fitness in Older Adults using Body Roundness Index and Waist Circumference
Source: Exerc Sport Mov. 2025 Dec 22;4(1):e00060. doi: 10.1249/ESM.0000000000000060 (PMC12721680; doi:10.1249/ESM.0000000000000060)
Supplement: Supplementary file 2 [file esam-4-e00060-s002.docx]

**Supplemental Content 2.** Standardized β coefficients from all four estimated cardiorespiratory fitness equations (eCRF).

| **Equation** | **Sex** | **Age** | **WC** | **BRI** | **SRPAS** | **IPAQ^++^** | **n** |
| --- | --- | --- | --- | --- | --- | --- | --- |
| eCRF1^IW^ | 0.75 (0.64, 0.87) | -0.31 (-0.42, -0.20) | -0.53 (-0.64, -0.41) | --- | --- | -0.03 (-0.08, 0.14) | 113 |
| eCRF2^SW^ | 0.60 (0.42, 0.78) | -0.29 (-0.46, -0.13) | -0.58 (-0.76, -0.40) | --- | 0.08 (-0.10, 0.25) | --- | 58 |
| eCRF3^SR^ | 0.48 (0.31, 0.65) | -0.30 (-0.47, -0.13) | --- | -0.57 (-0.74, -0.39) | 0.08 (-0.10, 0.25) | --- | 58 |
| eCRF4^IR^ | 0.61 (0.50, 0.72) | -0.30 (-0.41, -0.20) | --- | -0.51 (-0.62, -0.40) | --- | 0.02 (-0.10, 0.13) | 113 |

Standardized β coefficients and 95% confidence intervals for each variable used in the eCRF equations from Table 3, listed as standardized β (lower limit, upper limit). Dashed lines within rows indicate which variables are not included in that row’s equation. BRI (R), body roundness index; IPAQ^++^ (I), International Physical Activity Questionnaire with adjusted categories; SRPAS (S), Self-Report Physical Activity Survey; WC (W), waist circumference.
