# Supplementary material for: Nonexercise Equations for Cardiorespiratory Fitness in Older Adults using Body Roundness Index and Waist Circumference
Source: Exerc Sport Mov. 2025 Dec 22;4(1):e00060. doi: 10.1249/ESM.0000000000000060 (PMC12721680; doi:10.1249/ESM.0000000000000060)
Supplement: Supplementary file 3 [file esam-4-e00060-s003.docx]

**Supplemental Content 3.** Equations for estimating cardiorespiratory fitness (eCRF) from current analyses and established authors.

| Author | Equation | r | R^2^ | SEE | N |
| --- | --- | --- | --- | --- | --- |
| eCRF1^IW^ | 55.9104-0.2715*Age+7.192*(Sex)-0.222*WC+0.340*IPAQ^++^ | 0.79 | 0.63 | 6.18 | 58 |
| eCRF2^SW^ | 55.288-0.281*Age+7.270*Sex-0.216*WC+0.547*SRPAS | 0.79 | 0.63 | 6.49 | 58 |
| eCRF3^SR^ | 43.576-0.285*Age+5.824*Sex-1.723*BRI+0.563*SRPAS | 0.79 | 0.63 | 5.62 | 58 |
| eCRF4^IR^ | 44.112-0.281*Age+5.761*Sex-1.7466*BRI+0.281*IPAQ^++^ | 0.79 | 0.63 | 3.50 | 58 |
| Wier et al. (7) ^IW^ | 59.416 - 0.327*Age + 11.488*Sex - 0.266*WC+ 1.297*IPAQ | 0.81 | 0.66 | 4.80 | 2801 |
| Jurca et al. (14) ^SM^ | (18.07 -0.10*Age+2.77*Sex-0.17*BMI-0.03*RHR+SRPAS)*3.5 | 0.81 | 0.66 | 5.08 | 401 |

Reported Pearson’s r, R^2^, and the standard error of the estimate (SEE) in mL/kg/min are as listed by the original author for the two comparison equations and from *n*=58 EXTEND participants that completed the Self-Reported Physical Activity Survey (SRPAS). Age is in years. Sex is reported as male=1 and female=0. BMI (M), body mass index; BRI (R), body roundness index; IPAQ, International Physical Activity Questionnaire in three categories; IPAQ^++^ (I), IPAQ adjusted to six categories; RHR, resting heart rate (bpm); S, SRPAS; WC (W), waist circumference (cm).
