# Supplementary material for: Nonexercise Equations for Cardiorespiratory Fitness in Older Adults using Body Roundness Index and Waist Circumference
Source: Exerc Sport Mov. 2025 Dec 22;4(1):e00060. doi: 10.1249/ESM.0000000000000060 (PMC12721680; doi:10.1249/ESM.0000000000000060)
Supplement: Supplementary file 4 [file esam-4-e00060-s004.docx]

**Supplemental Content 4.** Comparison of equations when validated on BIKE participants.

| Equation | R (LL,UL) | R^2^ |
| --- | --- | --- |
| eCRF1^IW^ | 0.64 (0.37, 0.81) | 0.41 |
| eCRF2^SW^ | 0.65 (0.39, 0.81) | 0.42 |
| eCRF3^SR^ | 0.69 (0.44, 0.83) | 0.47 |
| eCRF4^IR^ | 0.69 (0.44, 0.84) | 0.47 |
| Wier et al. (7) ^IW^ | 0.67 (0.42, 0.83) | 0.45 |
| Jurca et al. (14) ^SM^ | 0.63 (0.35, 0.80) | 0.39 |

Equations from Supplemental Content 3 were validated on BIKE participants (n=32). CE = Σ(measured VO_2max_ – estimated VO_2max_)/n. Superscript letters are used to indicate which anthropometric measure and physical activity survey were used in each respective equation. eCRF, estimated cardiorespiratory fitness equation; I, International Physical Activity Questionnaire with adjusted categories; M, body mass index; R, body roundness index; S, Self-Report Physical Activity Survey; W, waist circumference.
