# Supplementary material for: Nonexercise Equations for Cardiorespiratory Fitness in Older Adults using Body Roundness Index and Waist Circumference
Source: Exerc Sport Mov. 2025 Dec 22;4(1):e00060. doi: 10.1249/ESM.0000000000000060 (PMC12721680; doi:10.1249/ESM.0000000000000060)
Supplement: Supplementary file 5 [file esam-4-e00060-s005.docx]

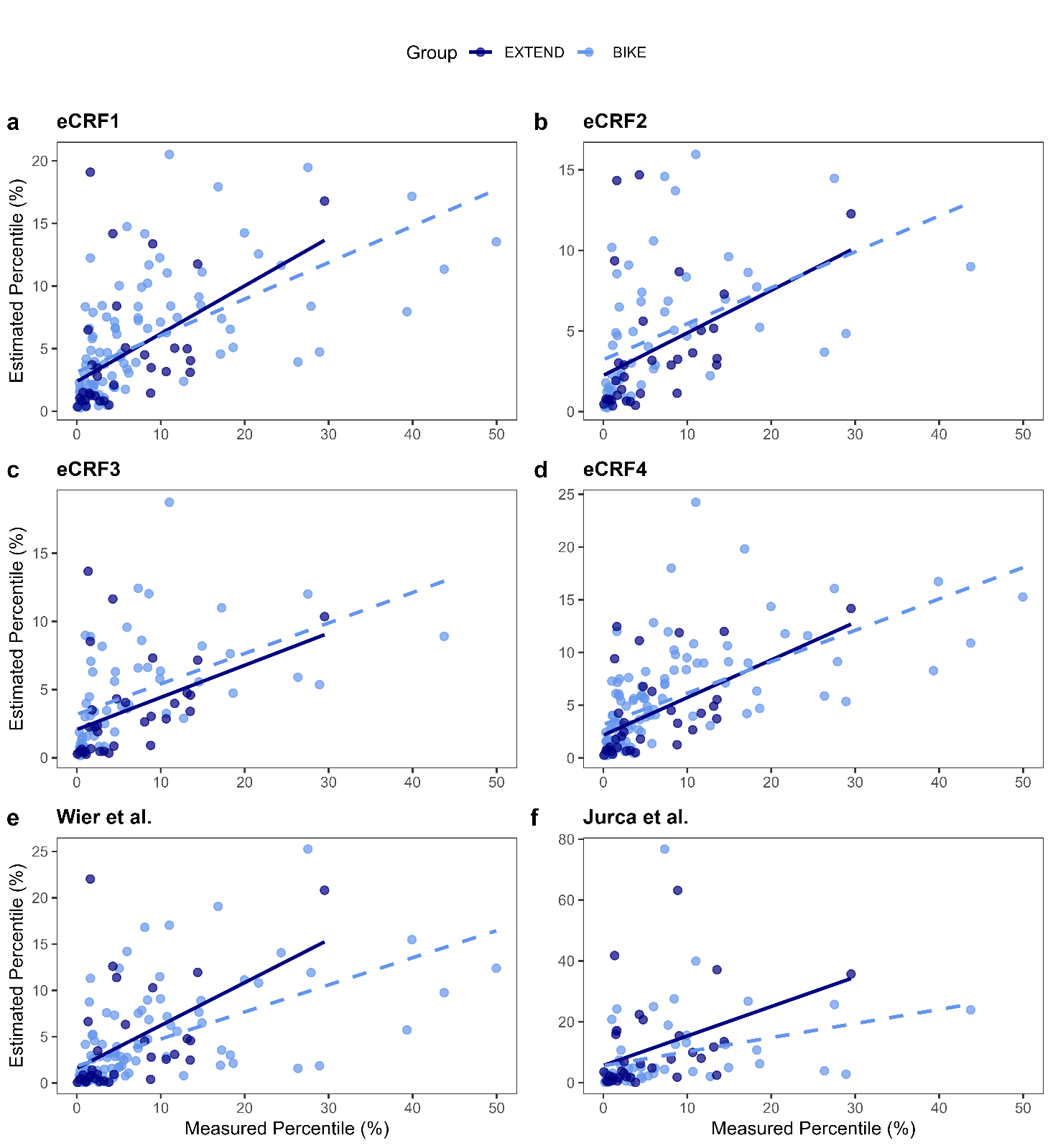


**Supplemental Content 5.** Scatterplots of measured cardiorespiratory fitness (CRF) percentiles and estimated CRF percentiles for each equation. a. Estimated CRF equation 1 (eCRF1). b. eCRF2. c. eCRF3. d. eCRF4. e. Wier et al. (7). f. Jurca et al. (14). Percentiles were calculated using a continuous, sex-specific model that is based on existing normative data from the American College of Sports Medicine for maximal oxygen consumption, allowing for the prediction of percentile rankings for individuals past 69 years old (Graves RS, Mahnken JD, Perea RD, et al. Modeling percentile rank of cardiorespiratory fitness across the lifespan. *Cardiopulm Phys Ther J*. 2015;26(4):108-13.).
