# Supplementary material for: Nonexercise Equations for Cardiorespiratory Fitness in Older Adults using Body Roundness Index and Waist Circumference
Source: Exerc Sport Mov. 2025 Dec 22;4(1):e00060. doi: 10.1249/ESM.0000000000000060 (PMC12721680; doi:10.1249/ESM.0000000000000060)
Supplement: Supplementary file 6 [file esam-4-e00060-s006.docx]

**Supplemental Content 6.** Distribution of age across categories of BMI and BRI.

| **BMI Category** | **Male** | | **Female** | | **BRI Category** | **Male** | | **Female** | |
| --- | --- | --- | --- | --- | --- | --- | --- | --- | --- |
|  | **Mean Age, yr** | ***n*** | **Mean Age, yr** | ***n*** |  | **Mean Age, yr** | ***n*** | **Mean Age, yr** | ***n*** |
| Underweight (<18.5) | -- | -- | 61 | 1 | Low (<3.74) | 66 | 8 | 64.4 | 25 |
| Normal (18.5-24.99) | 66.8 | 4 | 65.4 | 25 | Moderate (3.75-4.99) | 65.2 | 17 | 62.3 | 22 |
| Overweight (25-29.99) | 66 | 24 | 63.6 | 27 | High  (5-6.54) | 64.7 | 16 | 64.8 | 23 |
| Obese  (≥30) | 63.6 | 22 | 63.2 | 42 | Very High (≥6.55) | 64.2 | 9 | 64 | 25 |

Mean age in years from all participants in EXTEND and BIKE combined (*n*=145) across categories of body mass index (BMI) and body roundness index (BRI). Dashes indicate no available data. BMI categories from: World Health Organization. *Obesity: Preventing and Managing the Global Epidemic. WHO Technical Report Series 894*. Geneva: World Health Organization; 2000. 268 p. BRI categories from: Xu W, Zhang C, Feng W, Shi R. Association between body roundness index and lung function among U.S. adults. *BMC Public Health*. 2025;25(1):2429. doi: 10.1186/s12889-025-23649-5.
